# Mapping of Protein-Protein Interaction Sites in the Plant-Type [2Fe-2S] Ferredoxin

**DOI:** 10.1371/journal.pone.0021947

**Published:** 2011-07-08

**Authors:** Haruka Kameda, Kei Hirabayashi, Kei Wada, Keiichi Fukuyama

**Affiliations:** Department of Biological Sciences, Graduate School of Science, Osaka University, Toyonaka, Osaka, Japan; National Institute for Medical Research, Medical Research Council, London, United Kingdom

## Abstract

Knowing the manner of protein-protein interactions is vital for understanding biological events. The plant-type [2Fe-2S] ferredoxin (Fd), a well-known small iron-sulfur protein with low redox potential, partitions electrons to a variety of Fd-dependent enzymes via specific protein-protein interactions. Here we have refined the crystal structure of a recombinant plant-type Fd I from the blue green alga *Aphanothece sacrum* (*As*Fd-I) at 1.46 Å resolution on the basis of the synchrotron radiation data. Incorporating the revised amino-acid sequence, our analysis corrects the 3D structure previously reported; we identified the short α-helix (67-71) near the active center, which is conserved in other plant-type [2Fe-2S] Fds. Although the 3D structures of the four molecules in the asymmetric unit are similar to each other, detailed comparison of the four structures revealed the segments whose conformations are variable. Structural comparison between the Fds from different sources showed that the distribution of the variable segments in *As*Fd-I is highly conserved in other Fds, suggesting the presence of intrinsically flexible regions in the plant-type [2Fe-2S] Fd. A few structures of the complexes with Fd-dependent enzymes clearly demonstrate that the protein-protein interactions are achieved through these variable regions in Fd. The results described here will provide a guide for interpreting the biochemical and mutational studies that aim at the manner of interactions with Fd-dependent enzymes.

## Introduction

Ferredoxins (Fds) are small iron-sulfur proteins with low redox potentials that are involved in diverse electron-transfer systems [1]. The plant-type [2Fe-2S] Fds function not only in photosynthesis [2], where they transfer electron from photosystem I to Fd-NADP^+^ reductase (FNR), but also in electron transfer to such Fd-dependent enzymes as sulfite reductase, nitrite reductase, and Fd-thioredoxin reductase (FTR) [3]. It was well known that Fd is a hub redox protein, and therefore extensive studies on the structure and function have been made to solve the puzzle of how this small protein partitions electron to a variety of Fd-dependent enzymes [4].

The crystal structure of plant-type [2Fe-2S] Fd was first determined for the Fd from *Spirulina platensis*, a blue-green alga, revealing to have now known β-grasp motif or UB fold consisting of a four-stranded β-sheet with an α-helix packed across its face, in which a [2Fe-2S] cluster is located close to the molecular surface in the loop between the α-helix and the third β-strand [5]–[7]. The crystal structure of the present Fd from *Aphanothece sacrum* (sequence identity with *S. platensis* Fd is 70.7 %) was second to be determined, demonstrating that both Fds share a common overall structure and [2Fe-2S] cluster binding site [8], [9]. Many structure determinations followed plant-type [2Fe-2S] Fds from various organisms; these studies showed that they all have a common main-chain structure [10], [11], but revealed that a short peptide segment in *A. sacrum* Fd deviates greatly from that of the other Fds [12]–[17]; in *A. sacrum* Fd, the short second α-helix in the vicinity of the active center was missing.

Biochemical data have accumulated demonstrating that the plant-type Fd and each Fd-dependent enzyme form a weak complex [18] and it is believed that efficient electron transfer is achieved by the specific interactions between the two proteins [19], [20]. Intriguingly, the surface regions of a given Fd involved in such interactions differ, depending on the Fd-dependent enzymes. For example, the D66N/D67N mutant protein of maize Fd was less efficient in its interaction with FNR than the E93Q mutant, whereas this relationship was reversed in the reaction with sulfite reductase [21]. In addition, two crystal structures of Fd-FNR complexes showed that the manners of protein-protein interactions differed depending on leaf and root Fds [20], [22]. Importantly, the residues in *As*Fd-I that are assumed to be involved in the interactions with many Fd-dependent enzymes are included in the segment whose conformation greatly deviates from those of other Fds. The crystal structure of *As*Fd-I, reported more than two decades ago, was determined using the diffraction data collected at room temperature with a four-circle diffractometer and an in-house X-ray generator; the final R-value was 23% at 2.2 Å resolution, implying that substantial errors were present in the 3D structure. Moreover, after the crystal structure was reported, the amino-acid sequence was revised on the basis of the nucleotide sequence of the gene; one residue was missing between the 59th and 60th residues and glutamic acid residues at 31 and 59 should be both glutamine [23].

This paper presents the crystal structure of [2Fe-2S] *As*Fd-I refined at 1.46 Å resolution using synchrotron radiation data by incorporating the correct primary structure, allowing a revision of the 3D structure that was previously reported [8], [9]. Also taking into account that the asymmetric unit contains four molecules that are arranged in no symmetrical manner, we assessed the flexibility of peptide segments in the molecule from the diversity of the four 3D structures. We also compared 3D structures of *As*Fd-I with those of other Fds in order to view the distribution of variable/invariable segments in the plant-type [2Fe-2S]. Incorporating the biochemical results, along with the structures of the complexes with Fd-dependent enzymes so far reported, a general view has been emerged that the three flexible regions on the surface of the plant-type Fd are involved in the complex formation.

## Results and Discussion

### Crystallization, X-ray analysis and revised 3D structure

The holo-form of *As*Fd-I was successfully prepared by co-expressing the synthesized corresponding gene and the *isc* operon [24] in *E. coli*, followed by the hydrophobic and anion-exchange chromatography, whereas it was previously isolated from *A. sacrum* cells [25]. The crystals were grown under similar conditions that were reported earlier [26], which diffracted to better than 1.4 Å resolution using the synchrotron radiation at SPring-8. The present diffraction experiment at microfocus beamline of BL32XU permitted us to collect useful data at 100 K to 1.46 Å resolution. This data yielded a 3.4-fold increase in independent reflections over the number used for the previous analysis [12].

**Figure 1:**
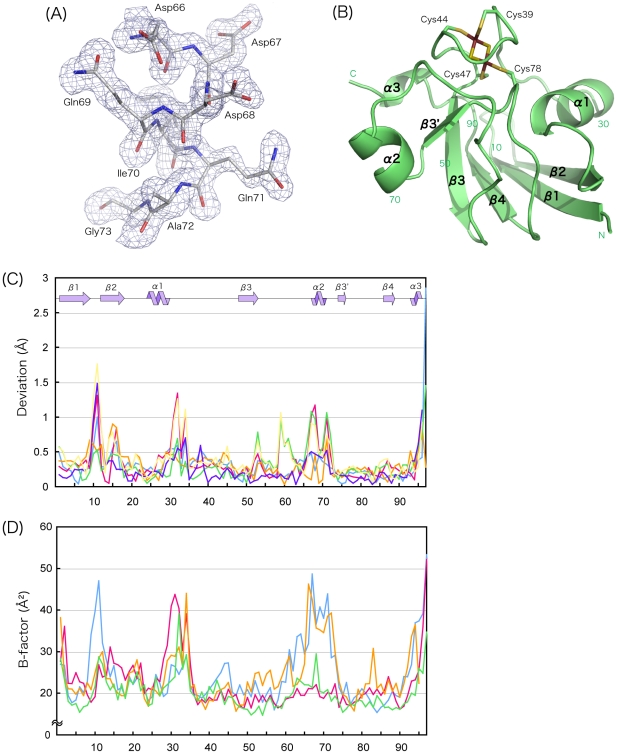
Revised structure of *As*Fd-I. (A) Omit *F*_o_–*F*_c_ map for segment 66–73 of the B molecule. The contour was drawn at 2σ. Segment 67–71 was clearly shown to adopt an α helix (α2). (B) Ribbon drawing of *As*Fd-I (B molecule). The [2Fe-2S] cluster and its ligand thiolate side-chains of cysteines are shown as sticks. (C) Deviations of the corresponding Cα atoms between pairs of molecules in the asymmetric unit along the sequence, where the deviations greater than 3 Å (the C-terminal Cα atoms of chain A - chain C, chain B - chain D, and chain C - chain D) are not shown. Blue, chain A - chain B; red, chain A - chain C; orange, chain A - chain D; green, chain B - chain C; purple, chain B - chain D; and yellow, chain C - chain D. The secondary structures are indicated above. (D) Temperature factors (Å^2^) of the Cα atoms: blue, chain A; red, chain B; orange, chain C; and green, chain D. The temperature factor above 60 Å^2^ (the Cα atom of the 96th residue of chain C) is not plotted. All structural figures were drawn with PyMOL [37].

This crystallographic refinement at 1.46 Å resolution (R/R_free_ = 0.187/0.224) revealed a prior misinterpretation of the electron density map, and allowed us to incorporate the correct amino-acid sequence [23] into the 3D structure; Ser60 that was missing earlier and the two glutamine residues at positions 31 and 59 that were incorrectly assigned to be glutamic acid were confidently fitted to the electron density map (Figure 1A). Notable revision of the main-chain conformation occurred in the segment 67–71, where it adopts a short α-helix, commonly observed in other plant-type [2Fe-2S] Fds. The Cα positions of the several residues including this segment in the present model deviate by 3–4 Å from those in the previous model (Figure S1). The other segments that have secondary structures were mostly unchanged; β1 and β2 were extended by a few residues (Figure 1B).

### Structural comparison of the four molecules in the asymmetric unit

Figure 1C shows the deviations of corresponding Cα atoms between pairs of the four molecules in the asymmetric unit when they are superimposed. Omitting two residues at the C-terminus, r.m.s. deviations are 0.32–0.50 Å. The iron-sulfur clusters and the protein atoms around the clusters superimpose fairly well onto each other in all four molecules. In contrast, in addition to the C-terminal residues, large deviations of the Cα atoms are seen in segments 9–12, 31–34, and 59–72. The former two segments correspond to the loop between β1 and β2 and the C-terminal end of α1 and the following loop, respectively, where multiple conformations are present. In segments 9–12 and 31–34, the conformations in pairs of A/D and B/C are similar. It is not unusual that these loop regions, as well as the terminal segments, can adopt variable conformations.

It is interesting to see how residues 67–72, the segment containing the α2 helix, deviate markedly with respect to each other. Here, the orientations of α2 are different; the helix in the C molecule is tilted by about 11° from the helix in the B molecule. The tilt of this helix occurs at Asp66 on one side and at Gly73 on the other side, where the torsion angles of these residues are diverse. That is, the conformational variability of segment 67–72 is caused by the diversity of α2 orientation, but not the deformation of α2. It is also apparent that the electron density for α2 and surrounding regions in the A and C molecules is low compared with the B and D molecules (Figure S2). In accordance with this observation, the temperature factors of the corresponding atoms of the A and C molecules are large compared with the B and D molecules (Figure 1D). These results can be interpreted as indicating that the intrinsically flexible α2 segment is fixed in some molecules by crystal-packing force. Indeed, the α2 helices whose main-chain atoms show high temperature factors are engaged in weak intermolecular interactions via water molecules in the crystal.

### Conserved variable/invariable regions in the plant-type [2Fe-2S] Fds

**Figure 2:**
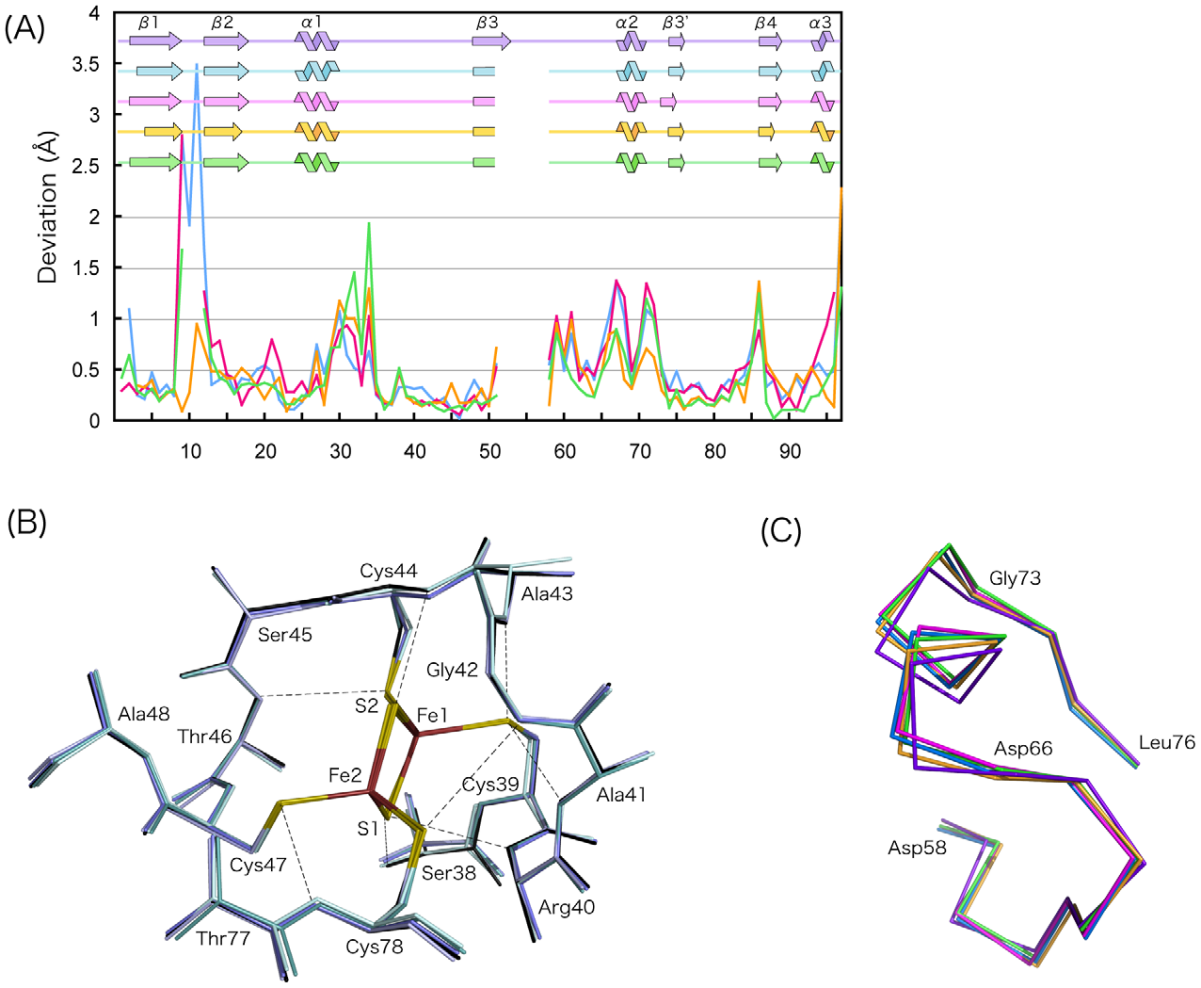
Comparison of the 3D structures of the plant-type [2Fe-2S] Fds. (A) Deviations of the corresponding Cα atoms between *As*Fd-I (chain B) and several plant-type Fds: blue, *Cyanidioschyzon merolae* Fd; red, *Mastigocladus laminosus* Fd; orange, *C. fusca* Fd; green, *Anabaena* PCC 7119 Fd. Because a Pro residue is inserted after the 56th residue (relative to other Fds), the deviations for its nearby residues are not given. (B) Structural comparison of the active centers of the plant-type [2Fe-2S] Fds. Only the structures determined at high resolutions (<1.5 Å) are included. For *As*Fd-I only the B molecule is drawn as representative. Broken lines indicate possible NH…S hydrogen bonds for *As*Fd-I. (C) Superposition of the Cα traces for segment 58-76 (in the sequence number of *As*Fd-I), which include the α2 helices. The colors used are the same as those in (A) except for *As*Fd-I (purple).

Comparisons of the 3D structures of the plant-type Fds from different organisms are shown in Figure 2A, where the deviations of the corresponding Cα atoms are shown for each pair of Fds. One notable finding is the high correlation between the deviations of the Cα atoms within the four molecules in the asymmetric unit of *As*Fd-I (Figure 1C) and between *As*Fd-I and the other Fds. As within the four molecules, the cluster and its immediate environment of *As*Fd-I superimpose fairly well with those of other Fds (Figure 2B). Consequently, the scheme of NH…S hydrogen bonding is conserved in the plant-type [2Fe-2S] Fds. The temperature factors of the atoms comprising the active site are generally small (Figure S3), suggesting that the active site region is rigid in all plant-type Fds.

On the other hand, the largest deviations among various Fds are seen in the loop between β1 and β2, the segment in which amino-acid sequences are least conserved; cyanobacterial Fds are shorter by two residues in this segment than Fds from plants [15]. In *Mastigocladus laminosus* Fd, the loop adopts different conformations in the two molecules in the asymmetric unit [16]. The other conformationally variable regions are seen at segments 29–34, the α2 helix and its preceding loop, and the C-terminus. Figure 2C shows a superposition of the segments of the α2 helices and their preceding loops in several Fds. In addition to the fact that the α2 helix and the preceding loop are flexible in *As*Fd-I, as described above, it was reported that in *Chlorella fusca* Fd the temperature factors of the atoms in this segment were high [15]. It is noteworthy that the 73rd residue, located at one side of the α2 helix, is conserved in most plant-type Fds as Gly, a residue that confers flexibility in peptide conformation. Its φ and ψ angles, even within *As*Fd-I, have wide range of values: 93.7° to 112.3° and −2.4° to 12.5°, respectively; these φ and ψ angles are in a generously allowed region in Ramachandran diagram. Diversity in the orientations of the α2 helices is achieved by this Gly residue. In other words, the 73rd position should be Gly in order for the α2 helix to be flexible. A superposition of the C-terminus of several Fds shows that conformational variability is caused partly by a swinging movement of α3 (Figure S4A). Segment 29–34 is in the loop following the α1 helix, and it may require no comment for this loop being flexible (Figure S4B). Taking the crystallographic results together, it may be regarded as being an intrinsic nature for all plant-type Fds that the active site and its environment is rigid whereas the four regions (I: the β1–β2 loop, II: the C-terminal end of α1 and the following loop, III: α2 helix and the preceding loop, and IV: the C-terminal) are flexible.

### Three flexible regions are involved in the protein-protein interactions

**Figure 3:**
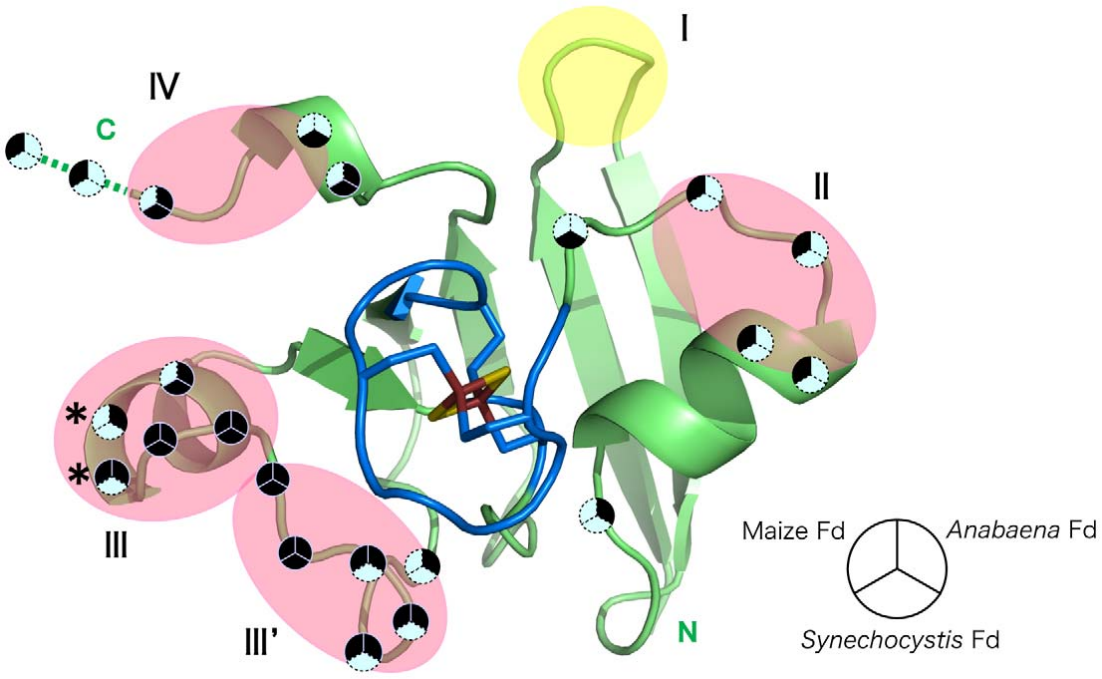
Overlay of the flexible regions on the residues involved in complex formation with Fd-dependent enzymes. The structure of the B molecule of *As*Fd-I is shown as a representative, where the two-residue extension of the C-terminus of another Fd is shown using a broken line. Regions II, III, III', and IV, shown in pink, are in the upper half and region I, shown in yellow, is the lower half of the molecule when viewed from the cluster side. The iron-sulfur cluster and its vicinity, which are rigid, are highlighted in different colors. Flags are placed on the sites corresponding to the residues of *As*Fd-I that are close to the Fd-dependent enzymes. For clarity, the flags in the immediate vicinity of the cluster (in blue) are not shown. When close intermolecular contacts are seen in each complex of *Anabaena* Fd-FNR (PDB code 1EWY), maize Fd-FNR (PDB code 1GAQ), and *Synechocystis* Fd-FTR and thioredoxin (PDB code 2PVO), the corresponding sections in the circles are shown in black. Stars, the residues pointed out by the mutational study for the interaction between maize Fd and FNR [21].

A ribbon drawing of the *As*Fd-I molecule viewed through the active center of the [2Fe-2S] cluster is shown in Figure 3, in which the four flexible regions assigned earlier (I–IV) are shaded. The β1–β2 loop (I) is located at the bottom of the molecule (opposite side of the active center), and thus it is unlikely that this loop is involved in the interaction with Fd-dependent enzymes. The remaining three regions (II-IV) are on the surface, and protrude in three directions from the active center. Figure 3 also shows the residues that are close to Fd-dependent enzymes in the structures of complexes that have been determined by X-ray crystallography [20], [22], [27]. It is notable that the residues involved in complex formation are mostly in the flexible regions of II–IV, although the manners of the complex formation differ greatly from each other depending on the source of the proteins. That is, in *Anabaena* Fd-FNR complex, the residues in regions III and IV face toward FNR [22], while in maize Fd-FNR complex, the residues in regions II and IV face toward FNR [20]. In the ternary complex of *Synechocystis* Fd with FTR and thioredoxin, the residues in regions III and IV face toward FTR [27]. Moreover, mutational study for maize Fd indicates that the 66th and 67th residues, which are in region III, are involved in interactions with some Fd-dependent enzymes [21]. Somewhat surprisingly, the C-terminal residues on the limited surface area of Fd, where acidic residues are clustered, are utilized in common for the protein-protein interaction.

In conclusion, the present analysis has revealed that in the plant-type Fds, the active center and its immediate environment are rigid and highly conserved, whereas the segments that encircle the active center are flexible. The structures of Fds in complex with Fd-dependent enzymes, and the biochemical data so far reported, clearly show that the residues that interact with Fd-dependent enzymes are localized on the flexible regions as well as in the active site. It is likely that the flexible nature of the segments contributes to fine-tuning of the specific interaction with a given enzyme, and that the main-chain conformations of these segments are perturbed upon complex formation. The overlap between the flexible regions and the protein-protein interaction sites in Fd presented here is reminiscent of the way in which antibodies recognize antigens through mobile loops [28]–[30]. The structures of spherical animal viruses also showed that the segments that interact with antibodies were in loops [31], [32]. Characterisitic seen in the plant-type Fd is that the segmental mobility arises not only from the flexible nature of the loops but also from the orientational diversity of the α2 helix. The ways for achieving specific interactions may be coming to be understood, but it is to be clarified more for other protein-protein complexes by analyzing the interface structures.

## Materials and Methods

### Protein expression and purification

A gene encoding *As*Fd-I protein was chemically synthesized based on the published nucleotide sequence [23] where the codon usage was optimized for the *E. coli* expression (Integrated DNA Technologies). The gene was cloned into the pET-21a(+) vector (Novagen) and co-expressed with the *isc* operon in *E. coli* strain C41(DE3) to produce holo-*As*Fd-I in high yield [23], [24]. The *As*Fd-I protein was purified using the HiPrep 16/10 Phenyl FF (low sub) column (GE Healthcare) followed by Mono Q HR 5/5 column (GE Healthcare) equipped with an ÄKTA explorer 10S system (GE Healthcare). The purified *As*Fd-I exhibited a UV-visible spectrum indistinguishable from what was previously reported [25] (Figure S5) and was concentrated by using a VIVASPIN filter (GE Healthcare).

### Crystallization, data collection and structure determination

Crystallization was attempted with the dialysis and vapor diffusion and oil batch methods using various precipitants. *As*Fd-I (about 100 mg/ml in 50 mM Tris-HCl (pH 7.8) and 400 mM NaCl) was eventually crystallized by dialyzing against a solution similar to the one used in a previously report [25]: 70–80% saturated ammonium sulfate, 100 mM Tris-HCl (pH 7.5) and 700 mM NaCl. Crystals were transferred to Paratone-N or a cryo-protectant solution containing 7.5% (v/v) glycerol and flash-cooled in a nitrogen gas stream at 100K. Diffraction data were collected using synchrotron radiation (λ = 1.000 Å) at the newly constructed microfocus beamline of BL32XU, which is equipped with a Rayonix MX225HE CCD detector, and also at BL38B1 (λ = 1.000 Å), which is equipped with ADSC Quantum 315 or CMOS detector [33] at SPring-8, Japan. Data were processed using the HKL2000 package. Because the data collected from the crystal in Paratone-N at BL32XU gave better diffraction statistics than any other data, that data set was used for the subsequent structural analysis.

Because the present crystal appeared to be nearly isomorphous with previously reported crystals (shrinkage of the unit-cell is due to the cryogenic temperature of this crystal), the coordinates of the four molecules in the asymmetric unit (PDB ID: 1FXI) were refined with rigid-body refinement, based on 25-3.0 Å resolution data using the CNS program. The electron density map calculated on the basis of the phases derived from the protein coordinates at this stage clearly located the [2Fe-2S] clusters. The model, which included the clusters, was further refined at 1.46 Å resolution via alternating cycles of simulated annealing, energy minimization and individual temperature-factor refinement, and the model revision with COOT [34]. The final R-factor and R_free_ were 18.7% and 22.4%, respectively. Statistics of the data collection and structure refinement are summarized in Table 1. Superposition and r.m.s. deviations of the structures were calculated using SSM superpose [35] in COOT and LSQMAN [36], respectively. Data Deposition: The atomic coordinates have been deposited in the Protein Data Bank, www.pdb.org (PDB ID code 3AV8).

**Table 1:** Statistic of the data collection and structure refinement.

Crystallographic data	
Space group	P4_1_
Unit cell dimensions (Å)	
a, b	92.0
c	46.3
Diffraction statistics^a^	
Wavelength (Å)	1.0000
Resolution (Å)	1.46 (1.51–1.46)
No. of observations	383,575
No. of unique reflections	66,810
Redundancy	5.7 (5.5)
Completeness (%)	99.0 (99.6)
Mean I_o_/σ (I)	17.0
* R*_sym_ b (%)	5.0 (38.3)
Refinement statistics	
R/R_free_ c (%)	18.7/22.4
Average B-factors	
Protein (Å^2^)	26.3
Water (Å^2^)	38.4
R.m.s. deviations from ideal values	
Bond length (Å)	0.027
Bond angle (°)	2.60
Ramachandran plot	
Most favored (%)	87.5
Additionally allowed (%)	11.6
Generously allowed (%)	0.9

a Values in parentheses correspond to the highest-resolution shell.

b R_sym_ = ∑*_hkl_*∑*_i_*|*I_i_*(*hkl*)-<*I*(*hkl*)>|/∑*_hkl_*∑*_i_I_i_*(*hkl*).

c R-factor = ∑*_hkl_*||*F*_o_(*hkl*)|-|*F*_c_(*hkl*)||/∑*_hkl_*|*F*_o_(*hkl*)|. R_free_ is the R-factor calculated for 5% of the data set not included in refinements.

## Supporting Information

Figure S1 **Superposition of the previous (PDB: 1FXI) and present models.** (A) A chain, (B) B chain, (C) C chain and (D) D chain. Left panels indicate the deviations of the corresponding Cα atoms between pairs of molecules, where the Cα atoms missing the corresponding pair and the Cα atoms whose deviations are greater than 4 Å (C-termini) are not shown. The right panels show superpositions of the previous (red) and present models (green).
(TIF)

Figure S2 **Electron density map for the α2 helix and nearby residues in** ***As*** **Fd-I.** (A) A molecule, (B) C molecule, and (C) D molecule. The contour was drawn at 2σ level.
(TIF)

Figure S3 **Temperature factors of the Cα atoms of various Fds.** *As*Fd-I in this study (purple trace), *Cyanidioschyzon merolae* Fd (blue trace), *Mastigocladus laminosus* Fd (red trace), *Chlorella fusca* Fd (orange trace), *Anabaena* PCC 7119 Fd (green trace).
(TIF)

Figure S4 **Superposition of (A) C-terminus and (B) the C-terminal end of the α1 helix and the following loop.** The same colors are used as in Figure 2C.
(TIF)

Figure S5 **UV-visible absorption spectra of the [2Fe-2S]** ***As*** **Fd-I.** Purified *As*Fd-I was dissolved in 50 mM Tris-HCl (pH 7.8) containing 400 mM NaCl. The spectrum was recorded at room temperature. Inset: SDS-PAGE analysis of *As*Fd-I used in this study. The gel was stained with Coomassie Blue.
(TIF)
