# Supplementary figures and images for: Mapping of Protein-Protein Interaction Sites in the Plant-Type [2Fe-2S] Ferredoxin
Source: PLoS One. 2011 Jul 8;6(7):e21947. doi: 10.1371/journal.pone.0021947 (PMC3132287; doi:10.1371/journal.pone.0021947)

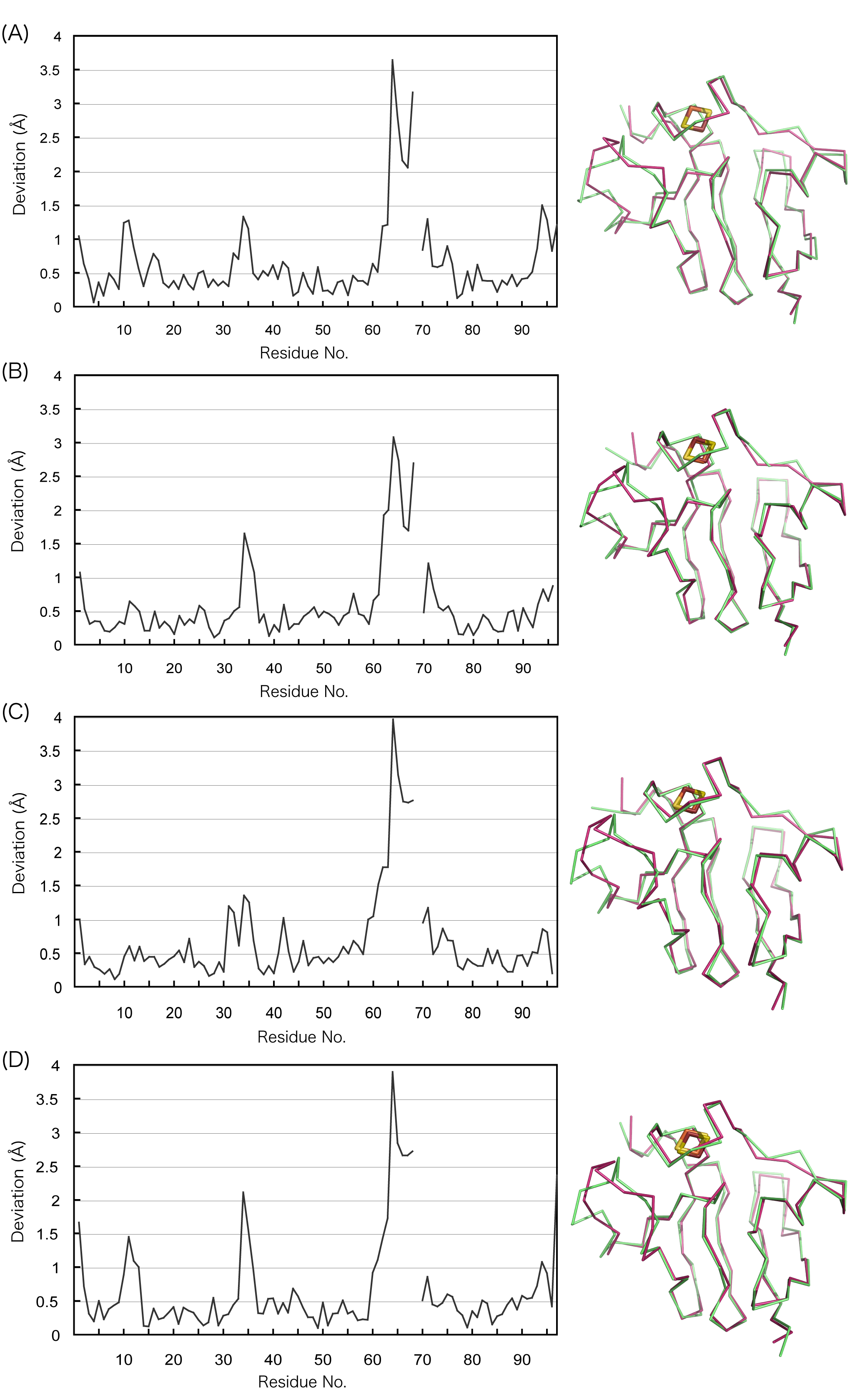

Supplement: Figure S1 — Superposition of the previous (PDB: 1FXI) and present models. (A) A chain, (B) B chain, (C) C chain and (D) D chain. Left panels indicate the deviations of the corresponding Cα atoms between pairs of molecules, where the Cα atoms missing the corresponding pair and the Cα atoms whose deviations are greater than 4 Å (C-termini) are not shown. The right panels show superpositions of the previous (red) and present models (green). (TIF) [file pone.0021947.s001.tif]

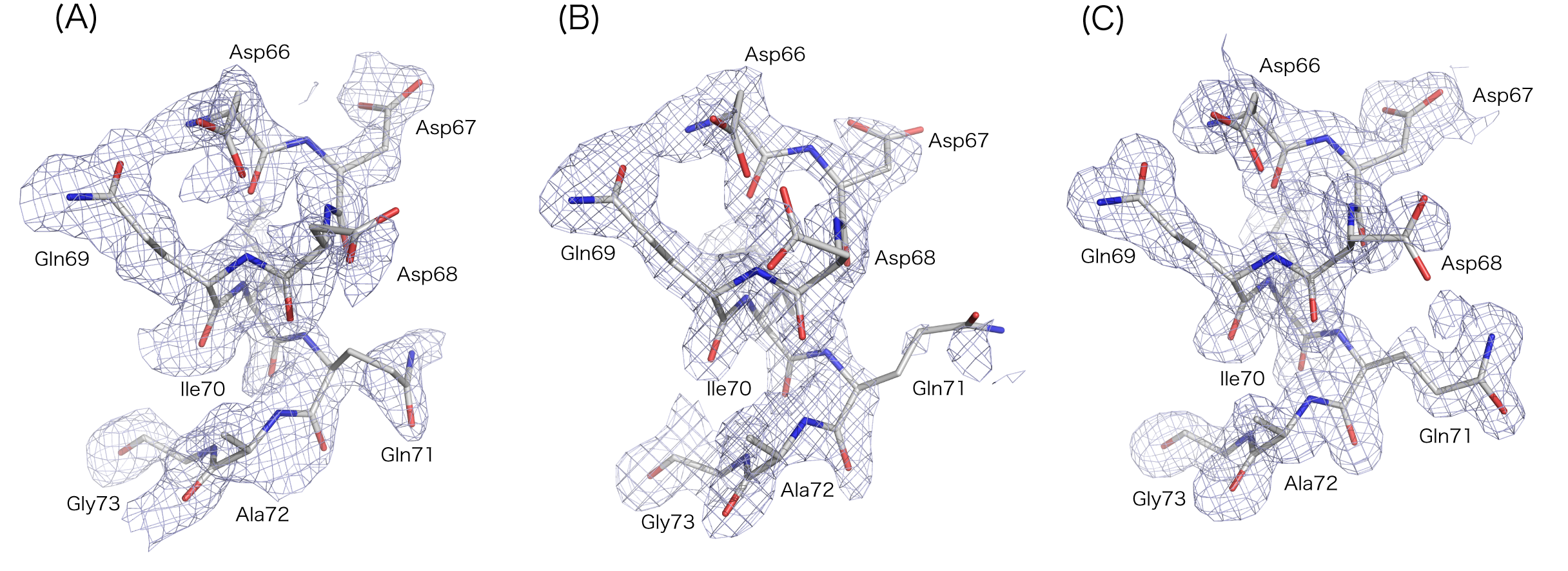

Supplement: Figure S2 — Electron density map for the α2 helix and nearby residues in As Fd-I. (A) A molecule, (B) C molecule, and (C) D molecule. The contour was drawn at 2σ level. (TIF) [file pone.0021947.s002.tif]

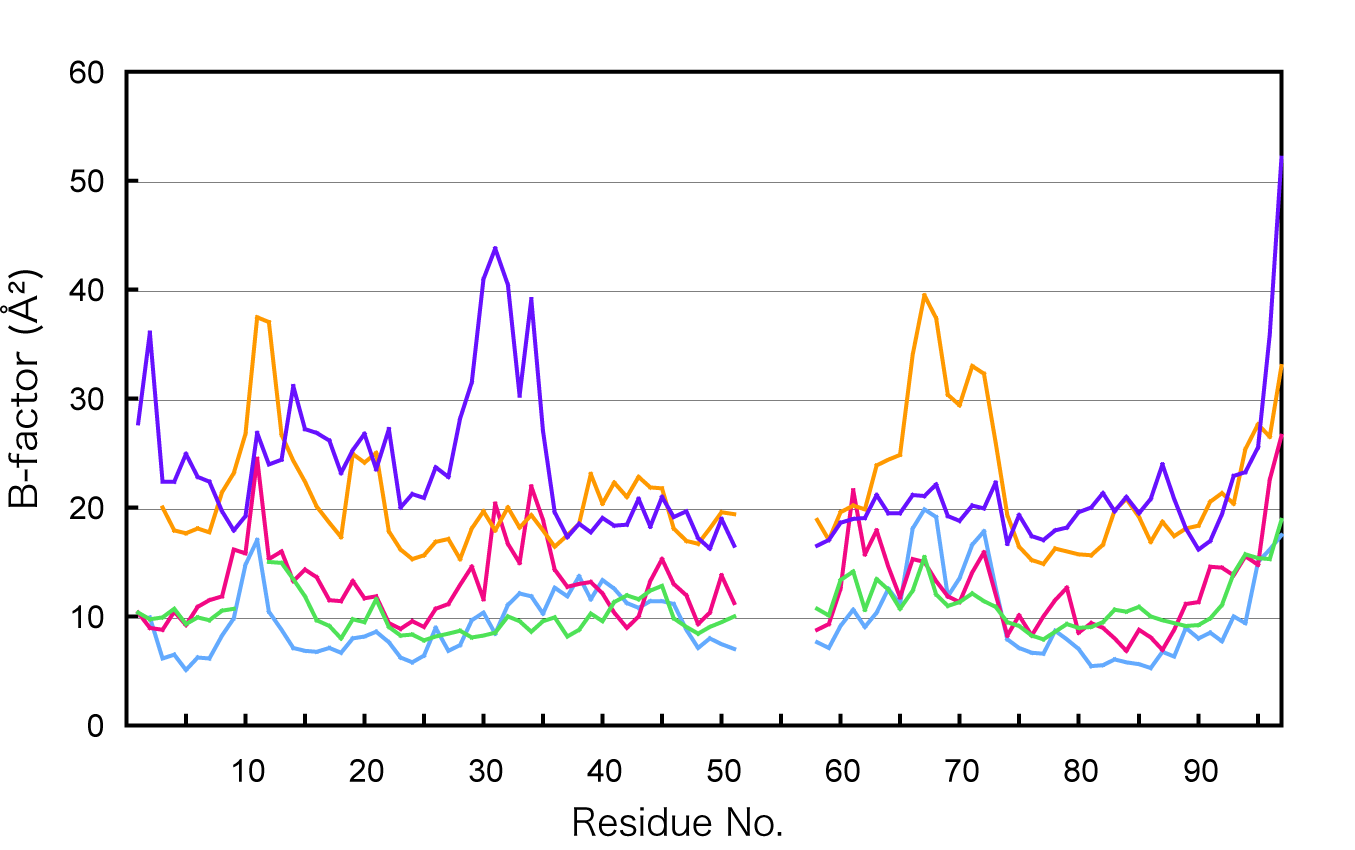

Supplement: Figure S3 — Temperature factors of the Cα atoms of various Fds. AsFd-I in this study (purple trace), Cyanidioschyzon merolae Fd (blue trace), Mastigocladus laminosus Fd (red trace), Chlorella fusca Fd (orange trace), Anabaena PCC 7119 Fd (green trace). (TIF) [file pone.0021947.s003.tif]

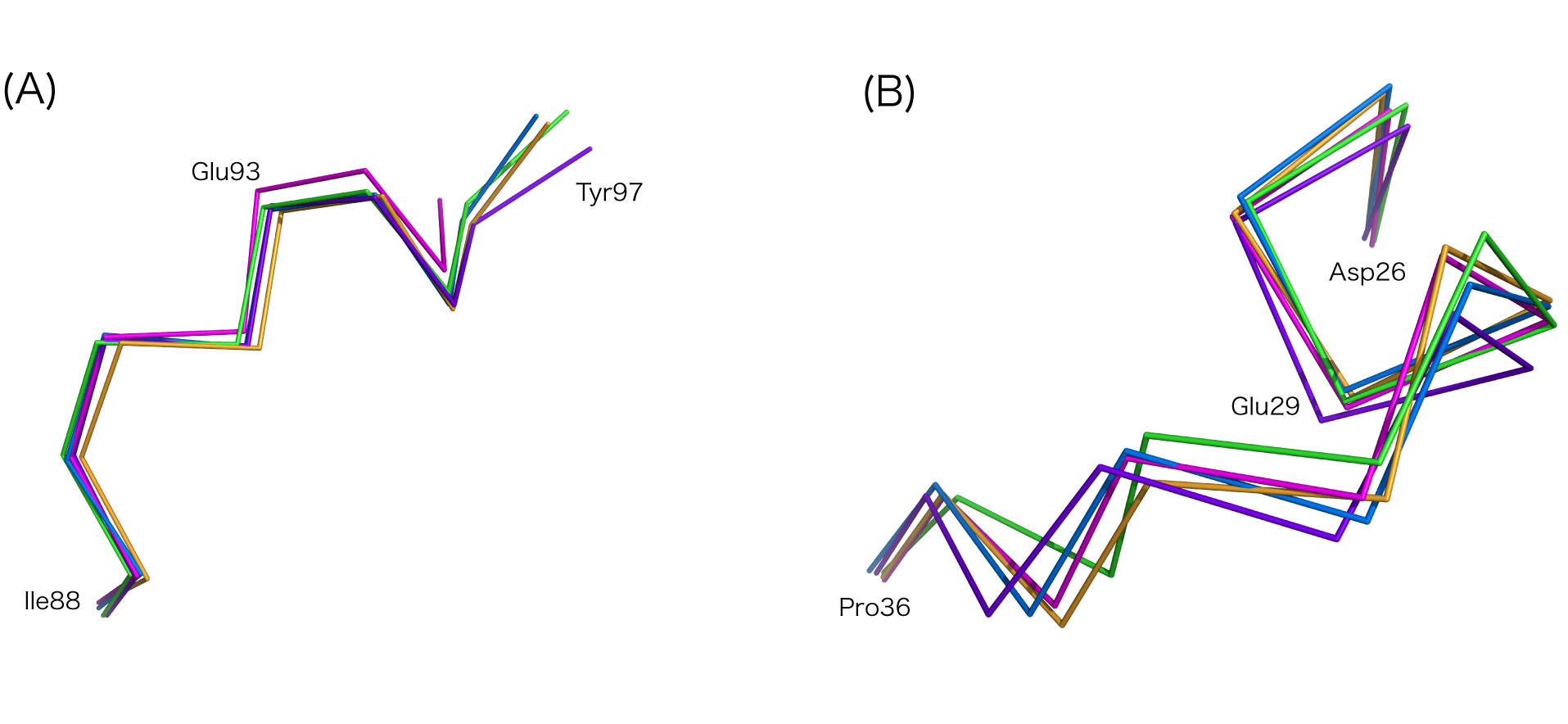

Supplement: Figure S4 — Superposition of (A) C-terminus and (B) the C-terminal end of the α1 helix and the following loop. The same colors are used as in Figure 2C. (TIF) [file pone.0021947.s004.tif]

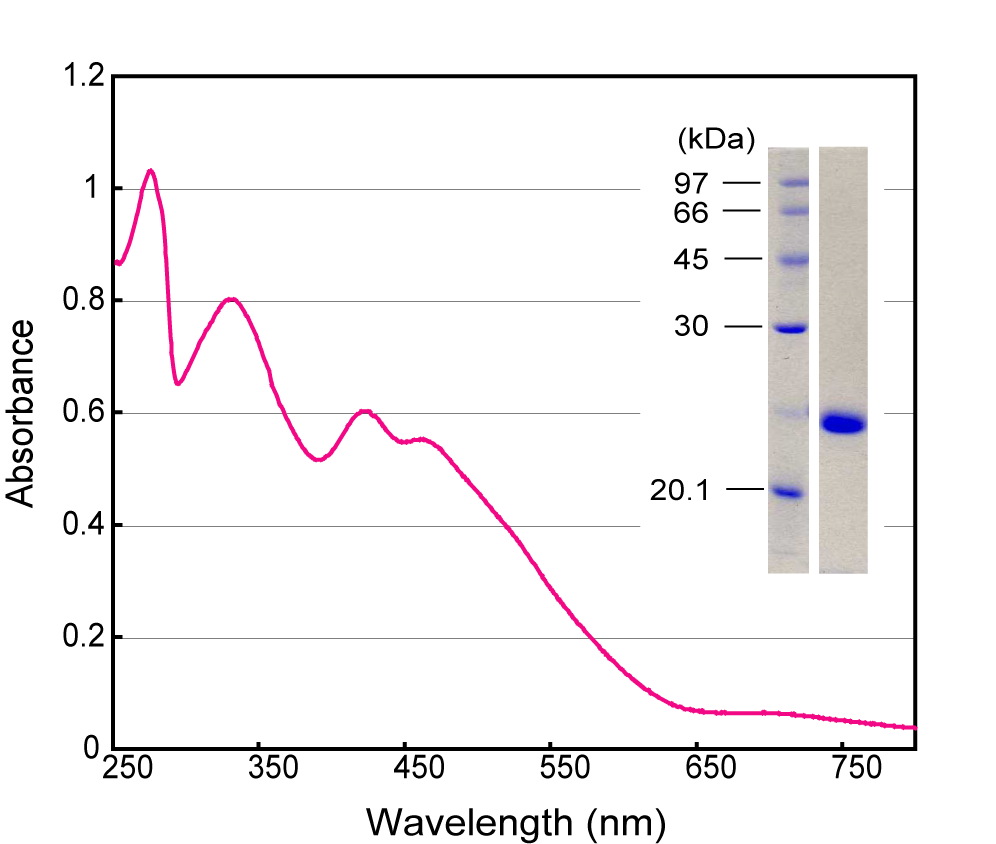

Supplement: Figure S5 — UV-visible absorption spectra of the [2Fe-2S] As Fd-I. Purified AsFd-I was dissolved in 50 mM Tris-HCl (pH 7.8) containing 400 mM NaCl. The spectrum was recorded at room temperature. Inset: SDS-PAGE analysis of AsFd-I used in this study. The gel was stained with Coomassie Blue. (TIF) [file pone.0021947.s005.tif]
